# Explaining the polarized macrophage pool during murine allergic lung inflammation

**DOI:** 10.3389/fimmu.2022.1056477

**Published:** 2022-12-20

**Authors:** Christina Draijer, Laura Florez-Sampedro, Catharina Reker-Smit, Eduard Post, Fransien van Dijk, Barbro N. Melgert

**Affiliations:** ^1^ GRIAC- Groningen Research Institute for Asthma and COPD, University Medical Center Groningen, University of Groningen, Groningen, Netherlands; ^2^ Department of Chemical and Pharmaceutical Biology, University of Groningen, Groningen, Netherlands; ^3^ Department of Molecular Pharmacology, University of Groningen, Groningen, Netherlands; ^4^ Department of Pharmacokinetics, Toxicology and Targeting, University of Groningen, Groningen, Netherlands

**Keywords:** M1 macrophages, M2 macrophages, alternatively activated, classically activated, asthma, alveolar macrophages, interstitial macrophages, YM1/Chi3l3

## Abstract

**Introduction:**

Differentially polarized macrophages, especially YM1+ and MHCII+ macrophages, play an important role in asthma development. The origin of these polarized macrophages has not been elucidated yet. We therefore aimed to investigate how proliferation, monocyte recruitment, and/or switching of polarization states contribute to this specific pool of polarized interstitial and alveolar macrophages during development of house dust mite (HDM)-induced allergic lung inflammation in mice.

**Methods:**

Male and female mice were first treated intranasally with PKH26 to label lung-resident macrophages and were then exposed to either HDM or phosphate-buffered saline (PBS) for two weeks. Different myeloid immune cell types were quantified in lung tissue and blood using flow cytometry.

**Results:**

We found that macrophage polarization only starts up in the second week of HDM exposures. Before this happened, unpolarized alveolar and interstitial macrophages transiently increased in HDM-exposed mice. This transient increase was mostly local proliferation of alveolar macrophages, while interstitial macrophages also contained unlabeled macrophages suggesting monocyte contribution. After two weeks of exposures, the number of interstitial and alveolar macrophages was similar between HDM and PBS-exposed mice, but the distribution of polarization states was remarkably different. HDM-exposed mice selectively developed YM1+ alveolar macrophages and MHCII-hi interstitial macrophages while nonpolarized macrophages were lost compared to PBS-exposed mice.

**Discussion:**

In this HDM model we have shown that development of a polarized macrophage pool during allergic inflammation is first dependent on proliferation of nonpolarized tissue-resident macrophages with some help of infiltrating unlabeled cells, presumably circulating monocytes. These nonpolarized macrophages then acquire their polarized phenotype by upregulating YM1 on alveolar macrophages and MHCII on interstitial macrophages. This novel information will help us to better understand the role of macrophages in asthma and designing therapeutic strategies targeting macrophage functions.

## Introduction

Macrophages are abundantly present in lung tissue and have many defensive and homeostatic functions (1). They can eliminate microbes and particles, coordinate tissue repair in case of damage, and downregulate inflammatory responses, all in a way that preserves gas exchange as much as possible. In order to maintain these many different functions, macrophages are known to have a spectrum of different polarization states to carry out the appropriate defensive and homeostatic functions (2). For instance, macrophages expressing high levels of interferon regulatory factor 5 (IRF5) and major histocompatibility complex class II (MHCII) have high antimicrobial capacity, while macrophages expressing high levels of chitinase 3-like 3 (YM1, mouse) and/or CD206 (human) promote wound healing, and macrophages expressing high levels of interleukin 10 (IL-10) have anti-inflammatory activity (3–5). We have previously shown that in healthy lung tissue of both humans and mice, IL10+ macrophages predominate over both IRF5+MHCII-hi and YM1+CD206+ macrophages (6–10). However, in people or mice with asthma/allergic lung inflammation their proportions change dramatically to an overabundance of both IRF5+MHCII-hi and YM1+CD206+ macrophages and fewer IL10+ macrophages (6–8, 10). It is currently unknown how and from which cells these different macrophage polarization states develop in the lung during allergic lung inflammation.

In addition to different polarization states, lung macrophages can also be divided into two populations depending on their location, i.e. alveolar and interstitial macrophages (1). These two different populations have different functions, origins, and different ways of replenishment. Alveolar macrophages have embryonic progenitors and are maintained throughout life through local proliferation (11–13). This does not necessarily exclude monocytes as precursors for alveolar macrophages since depletion of lung macrophages followed by adoptive transfer of bone marrow, monocytes, or Ly6c-low expressing monocytes showed that alveolar macrophages can be replenished from bone marrow during steady state conditions (14–16). Interstitial macrophages derive from yolk sac macrophages early during development and a large proportion of those are later replaced by bone marrow-derived interstitial macrophages that are maintained by circulating monocytes (17, 18). Yet how these two different types of macrophages contribute to the emergence of the differentially polarized subsets in asthma is unclear. Previous work by Zaslona et al. has shown that maintenance of the alveolar macrophage pool in a mouse model of asthma mostly depends on local proliferation of resident macrophages rather than on recruitment of monocyte-derived macrophages (19). However, this study focused on alveolar macrophages and interstitial macrophages were not studied. In addition, no study has addressed how differentially polarized macrophages develop in asthma, i.e. from nonpolarized alveolar and/or interstitial macrophages, through proliferation, and/or from incoming monocytes?

Since the available evidence suggested local proliferation being the main source of macrophages during allergic inflammation (19), we hypothesized that the different polarization states found in allergic lung inflammation would also originate through local proliferation and concurrent polarization. To investigate this hypothesis, we used a mouse model of house dust mite (HDM)-induced allergic lung inflammation to study the kinetics of lung-resident macrophages after allergen exposure. We labeled lung resident macrophages with the carbocyanine fluorescent dye PKH26 at the start of the experiment to track them in lung tissue during two weeks of development of HDM-induced lung inflammation. Using this model, we found that the process of YM1-polarization happens in tissue-resident alveolar macrophages and that replenishment through proliferation happens before polarization. In addition, we found that interstitial macrophages selectively upregulate MHCII and that these develop from incoming monocytes.

## Materials and methods

### Animals

Male and female wild-type (BALB/cOlaHsd) mice aged 6-8 weeks were purchased from Harlan (Horst, The Netherlands). The mice were housed in groups of 4 with ad libitum access to water and food. The Institutional Animal Care and Use Committee of the University of Groningen approved these experiments (application number 6272G), which were performed under strict governmental and international guidelines.

### *In vivo* labeling of macrophages and monocytes

The PKH26 red fluorescent cell linker kit for phagocytic cell labeling (Sigma-Aldrich, Zwijndrecht, The Netherlands) was used to label macrophages in the lung. PKH26 was prepared according to the manufacturer’s instructions. 75 ul of 13 μM PKH26 was intranasally administered to mice that were under isoflurane anesthesia. To label blood monocytes, mice were intra peritoneally (i.p.) injected with 200 μl of 2 mg 5-bromo-2’-deoxyridine (BrdU, Sigma) 18 hours before each HDM/PBS exposure. Unfortunately, the BrdU staining was not detectable using flow cytometry and cells were not analyzed for BrdU incorporation.

### House dust mite protocol

Mice (4 males and 4 females per group) were exposed intranasally to whole body HDM extract (Dermatophagoides pteronyssinus, lot number 140153, Greer laboratories, Lenoir, USA containing 7 EU/mg endotoxin) or phosphate-buffered saline (PBS) according to a 14-day model (10). Briefly, mice were anesthetized with isoflurane and received a sensitization dose of 100 μg HDM in 40 μl PBS on day 0 and were challenged with 10 μg HDM in 40 μl PBS on day 7-11 to induce allergic lung inflammation. Mice exposed to 40 μl PBS according to the HDM schedule served as healthy controls. On the day of sacrifice, mice were anesthetized with isoflurane and first blood was removed by heart puncture in a heparinized tube. Then the abdomen and chest cavity were exposed and the caudal vena cava was cut. The pulmonary circulation was subsequently flushed with 10 ml of PBS via the right ventricle to remove blood and circulating immune cells. The right lung was collected for flow cytometry and the left lung was collected for histology.

### Experimental design

**Figure 1:**
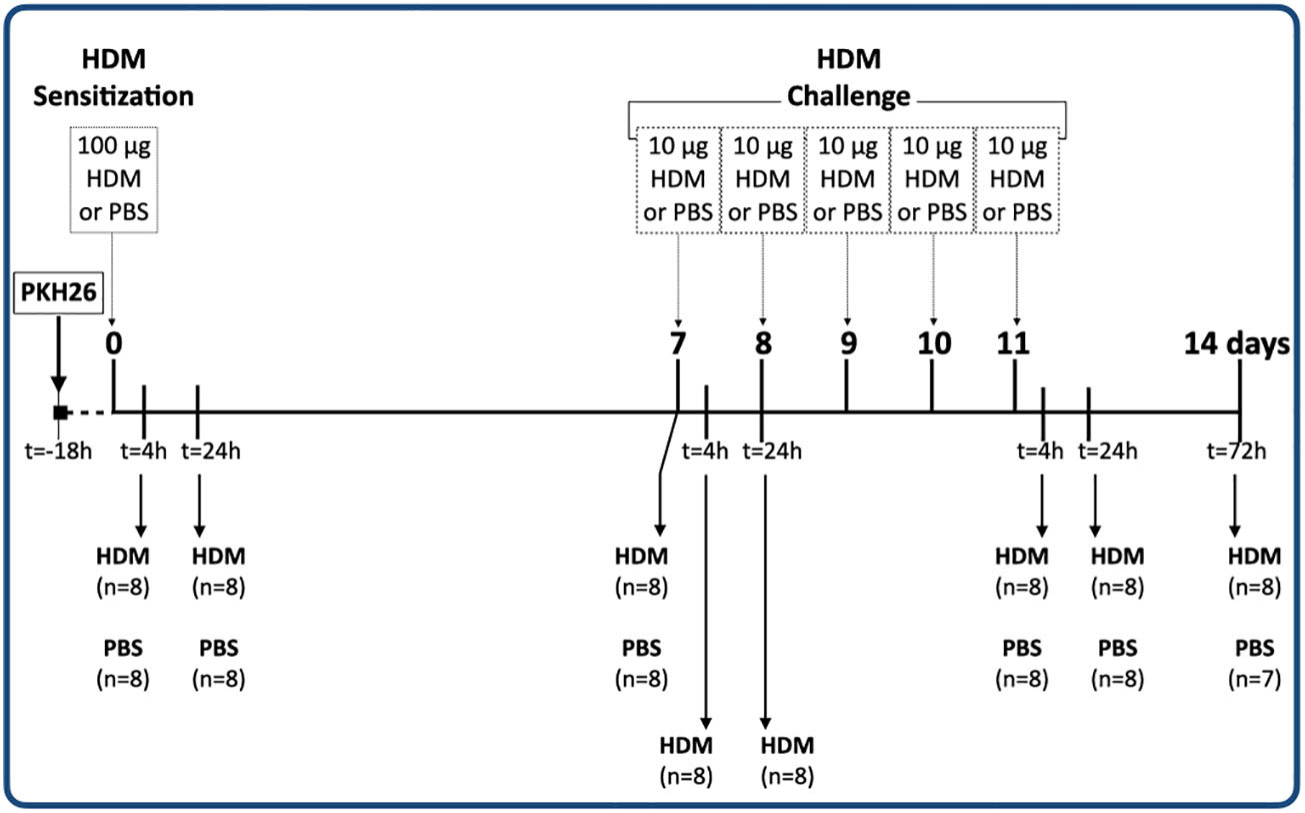
Experimental design of the study.

The experimental design of study is depicted in **Figure 1**. A total of 120 mice were included in this study, but the tissue sample of 1 mouse (male PBS 14 days) was lost during sample preparation. Each group was sacrificed at a specific time point within the 14-day HDM model. The experiments were done in a staggered manner and therefore each experimental group consisted of mice sacrificed on different days to exclude the influence of batch effects.

18 hours after *in vivo* labeling with PKH26, all groups received the sensitization dose of 100 μg HDM or PBS (healthy controls). Three groups were sacrificed 4 hours (day 0.2), 1 and 7 days after administering 100 μg HDM or PBS (healthy controls). Two more groups were sacrificed 4 hours (days 7.2) and 1 day (day 8) after administering the first challenge with 10 μg HDM. There were no healthy control groups included at these time points. The last groups of mice were sacrificed 4 hours (day 11.2), 1 (day 12) and 3 days (day 14) after the last HDM/PBS administration respectively.

### Lung digestion

The right lung lobe was minced and incubated in RPMI 1640 medium (Lonza, Verviers, Belgium) supplemented with 10% fetal calf serum (Lonza), 0.7 mg/ml collagenase A (Roche Applied Science, Almere, Netherlands) and 10 μg/ml DNAse I (grade II from bovine pancreas, Roche Applied Science) for 45 min at 37°C in a shaking water bath. After digestion, the lung tissue was passed through a 70 μm-pore nylon strainer (BD Biosciences, Breda, Netherlands) to obtain single cell suspensions. A 2-min incubation with 10 times diluted Red Blood Cell lysis buffer (Biolegend, Fell, Germany) was performed to lyse contaminating erythrocytes, followed by centrifugation through 70 μm pore strainer caps. Cells were counted using a BD FACS array (BD Biosciences) and were ready for flow cytometry staining.

### Flow cytometric analysis

Single lung cell suspensions were stained for macrophages, dendritic cells, eosinophils, neutrophils, and monocytes using a mix of antibodies for flow cytometry. The antibodies used are shown in **Table 1** and were used and tested before (20, 21).

**Table 1:** Information of antibodies used to stain lung or blood leukocytes.

Antibody	Fluorophore	Company	Cat number
Live/dead dye	eFluor 506	ThermoFisher	65-0866-14
anti-CD64	PE/Cy7	Biolegend	139313
anti-CD68	PerCPcy5.5	Biolegend	137010
anti-YM1	Biotinylated	R&D Systems	BAF2446
–	Streptavidin BUV395	BD Biosciences	564176
anti-MHC class II	APC/Cy7	Biolegend	107628
anti-CD206	Alexa Fluor 647	Biolegend	141712
anti-GR1	Alexa Fluor 700	Biolegend	108422
anti-CD11c	Brilliant Violet 785	Biolegend	117336
anti-CD11b	Brilliant Violet 570	Biolegend	101233
anti-Ly6C	Alexa Fluor 488	Biolegend	128022

Approximately 1x10^6^ lung cells were incubated with the appropriate extracellular antibody mix containing 1% normal mouse serum for 30 min on ice in the dark. The blood was first 1:1 diluted at room temperature in PBS, centrifuged and then incubated with the extracellular antibody mix at room temperature. After subsequent washing of the cells with PBS supplemented with 2% FCS and 5 mM EDTA (PFE), cells were incubated with the live/dead dye for 30 min on ice (lung cells) and room temperature (blood). After that, cells were washed twice with PFE, and the blood cells were incubated for 30 min at room temperature in freshly prepared FACS lysing buffer (eBioscience) and then washed twice with permeabilization buffer. Next, all lung cells and white blood cells were incubated for 15 min with fixation and permeabilization buffer (eBioscience) at room temperature, and then washed twice with permeabilization buffer. Subsequently, all cells were incubated with 0.3 mg/mL DNAse I (Roche) for 1 hour at 37°C in the dark and afterwards washed with permeabilization buffer. Cells were then incubated with the intracellular antibody mix containing 1% normal mouse serum for 30 min on ice in the dark followed by a 30-min incubation with streptavidin-Brilliant Ultra Violet 395 on ice. Afterwards cells were washed twice with permeabilization buffer and resuspended in PFE and kept in the dark on ice until flow cytometric analysis. The fluorescent staining was measured on an LSR-II flow cytometer (BD Biosciences) and data were analyzed using FlowJo Software (Tree Star, Ashland, USA).

To assess the quality of data collection, each sample was analyzed using the Flowjo plugin FlowAI version 2.3.1 (22). Setting used were: all checks for anomalies, a second fraction FR of 0.1, an alpha FR of 0.01, 3 maximum changepoints with 200 as a changepoint penalty, and both sides of the dynamic range were checked. Only events meeting the quality critera (good events) were used for further processing. Since macrophages seldom cluster in discrete populations, we first manually gated based on accepted strategies in literature and the markers used to identify each cell population are shown in **Table 2**. We then used Flowjo plugin ezDAFi version 0.5 (23), which automatically converts 2-dimensional manual gates into natural-shaped populations in the multidimensional space. This allows for improved cell population identification and proportion quantification as all color-dimensions are considered when assigning cells to particular gates. We clustered with self-organizing maps at a value of 3. Examples or our gating strategies and resulting populations can be found in the **Supplementary Data, Supplementary Figures 1A-C**.

**Table 2:** Markers used to characterize different populations of myeloid cells.

Cell type	Markers
Macrophages	CD68^hi^
Alveolar macrophages	CD68^hi^CD11c^hi^CD11b^low^
Interstitial macrophages	CD68^hi^CD11c^var^CD11b^var^
Nonpolarized macrophages (MHCII^lo^ YM1^lo^)	CD68^hi^MHCII^var^YM1^neg^
MHCII^hi^ macrophages (a.k.a. M1)	CD68^hi^MHCII^hi^YM1^neg^
YM1^+^ macrophages (a.k.a. M2)	CD68^hi^MHCII^low^YM1^+^
Neutrophils	CD68^neg^GR1^hi^
Eosinophils	CD68^neg^CD11c^low^MHCII^neg^
Dendritic cells	CD68^low^MHCII^+^CD11b^+^CD11c^+^
Tissue monocytes	CD68^low^CD11b^+^MHCII^neg^
Tissue Ly6C^high^ (Ly6Chi) monocytes	CD68^low^CD11b^+^MHCII^neg^Ly6C^hi^
Tissue Ly6C^low^ (Ly6Clo) monocytes	CD68^low^CD11b^+^MHCII^neg^Ly6C^low^
Blood monocytes	SSC^low^CD11b^+^GR1^low^
Blood Ly6C^high^ (Ly6Chi) monocytes	SSC^low^CD11b^+^GR1^low^Ly6C^hi^
Blood Ly6C^low^ (Ly6Clo) monocytes	SSC^low^CD11b^+^GR1^low^Ly6C^low^

### Lung histology

The left lung was carefully filled with 50% Tissue-Tek® O.C.T.™ compound in PBS (Sakura, Finetek Europe B.V., Zoeterwoude, The Netherlands) through a cannula inserted in the trachea. It was partly fixed in a zinc-containing buffer (JB fixative (24)) and embedded in paraffin and partly frozen for histology.

Expression of different macrophage markers and proliferation was determined in 3 μm paraffin sections or 4 μm frozen sections using standard immunohistochemical procedures. A staining for CD68 was done on frozen sections by fixing in acetone for 10 min and rehydration in PBS. This was followed a 1-hour incubation at room temperature of anti-murine CD68 (1:250, Serotec, UK) in PBS with 5% normal mouse serum. After washing with PBS, endogenous peroxidases were quenched by incubation in 0.3% H2O2 in methanol. After washing away this solution, sections were subsequently incubated with goat anti-rat immunoglobulins labeled with horse radish peroxidase (1:500, ThermoFisher, The Netherlands) and rabbit anti-goat immunoglobulins labeled with horse radish peroxidase (1:500, ThermoFisher) in PBS with 5% normal mouse serum for 30 min each and with washing in between. The staining was then visualized using a Novared substrate kit (Vector Laboratories, the Netherlands) according to the instructions of the kit. Sections were dehydrated and mounted using non-aqueous mounting medium.

YM1 (1:400, goat-anti-mECF-L, R&D Systems, UK) was similarly visualized on 3 μm paraffin sections that were first deparaffinized and rehydrated and incubated overnight in 10 mM citrate buffer pH 6 with 0.05% Tween-20. As a secondary antibody, rabbit anti-goat immunoglobulins labeled with horse radish peroxidase (1:500, ThermoFisher) in PBS with 5% normal mouse serum was used. The staining was visualized as described above for CD68.

The antibody against YM1 was also used in a double staining for K167 using immunohistochemical procedures described before (20). In short, 3 μm paraffin sections were treated as described above for the YM1 single staining. Sections were first incubated with goat anti-YM1 (1:400) in PBS with 5% normal mouse serum for 1 hour at room temperature. After washing with PBS, sections were then incubated for 1 hour at room temperature with rabbit anti-Ki67 (1:500, Abcam, Milton, UK) in PBS with 5% normal mouse serum. After washing with PBS, endogenous peroxidases were quenched by incubation in 0.3% H_2_O_2_ in methanol. After washing away this solution, sections were subsequently incubated with donkey anti-goat immunoglobulins labeled with horse radish peroxidase (1:500, ThermoFisher) and goat anti-rabbit immunoglobulins labeled with alkaline phosphatase (1:50, ThermoFisher) in PBS with 5% normal mouse serum for 30 min each and with washing in between. Ki67 staining was then visualized using a BCIP/NBT substrate kit (Vector Laboratories) followed by visualization of YM1 with a Novared substrate kit (Vector Laboratories) according to the instructions of the kits. Sections were then dehydrated and mounted using non-aqueous mounting medium.

This double staining for Ki67 and YM1 was quantified by counting YM1-positive cells and YM1-and Ki67-double positive cells in randomly selected areas of the section. Numbers were corrected for the total surface area of lung tissue as measured by Aperio ImageScope viewing software 11.2.0.780 (Aperio, USA).

### Statistical analysis

Data were found to be normally distributed with visual inspection of QQ plots and are represented as geometric mean with standard error of the mean. Curves of HDM-exposed mice and PBS-exposed mice were inspected visually and only time points that showed a relevant difference between PBS and HDM were tested using a Student’s t test per time point. Due to IACUC-enforced limitations, we had no matching PBS-exposed groups at days 7.2 and 8 and we needed to compare the HDM-exposed groups of those time points with the PBS-exposed group at day 7. Since this then involved comparing multiple groups, we used a one-way ANOVA with Sidak’s correction for multiple testing when we visually detected relevant differences on days 7.2 and 8 in HDM-exposed mice compared to PBS-exposed mice on day 7. For expression levels of different markers on interstitial and alveolar macrophages, we compared mean fluorescence intensity (MFI) values of each marker on either type of macrophages on day 11.2 of the model. Expression levels on alveolar versus interstitial macrophages were compared using a paired one-way ANOVA with a Sidak’s correction for multiple testing because these cells were isolated from the same animals. Expression levels on alveolar or interstitial macrophages in PBS-exposed animals versus HDM-exposed animals were compared using an unpaired one-way ANOVA with a Sidak’s correction for multiple testing because these cells were isolated from different animals. P-values <0.05 were considered to be statistically significant.

## Results

### HDM exposure induces allergic lung inflammation

To investigate the degree of allergic lung inflammation induced by exposure to HDM, we first studied numbers of immune cells that can infiltrate lung tissue from blood, i.e. eosinophils, neutrophils, dendritic cells, and monocytes during the course of our model (identification of these cells was done according to the gating strategies shown in the **Supplementary Data, Supplementary** **Figures 1A, B**).

**Figure 2:**
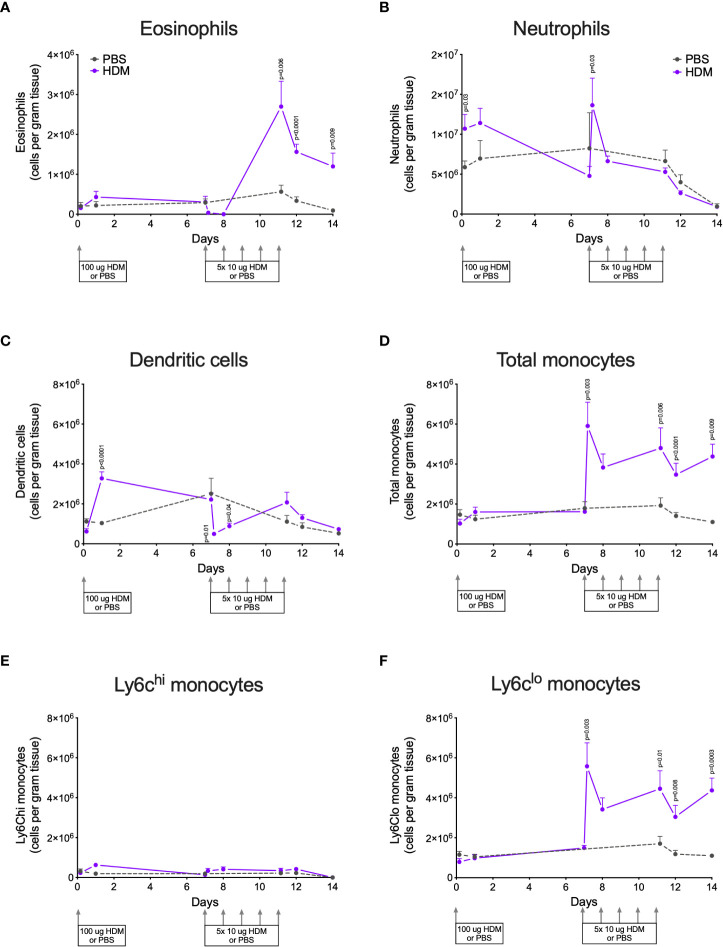
Numbers of **(A)** eosinophils, **(B)** neutrophils, **(C)** dendritic cells, **(D)** total monocytes, **(E)** Ly6Chi monocytes, and **(F)** Ly6Clo monocytes in lung tissue of house dust mite (HDM)-exposed and phosphate-buffered saline (PBS)-exposed mice at different time points. The differences between HDM and PBS per time point were tested using a Student’s t test. Day 7.2 and day 8 of HDM were compared to day 7 of PBS with a one-way ANOVA with Sidak’s correction for multiple testing because these mice did not have matching PBS-exposed mice. P<0.05 was considered significant. Per time point the geometric mean and 95% confidence intervals per group are shown (4 males and 4 females per group, except for PBS 14 days that had 3 males and 4 females).

HDM exposure resulted in significantly higher numbers of eosinophils in lung tissue compared to PBS exposure (**Figure 2A**). Infiltration of eosinophils into lung tissue was not apparent after the first (sensitization) dose of HDM but started later during the second week of HDM challenges.

HDM exposure also induced higher numbers of neutrophils in lung tissue as compared to PBS exposure (**Figure 2B**), although the kinetics of neutrophil infiltration were different from the kinetics of eosinophils. Significant neutrophil infiltration was seen within one day after the sensitization dose and the first challenge dose of HDM compared to PBS exposure.

The presence of dendritic cells in lung tissue followed yet another pattern: significantly higher numbers were found one day after HDM sensitization and significantly fewer within one day of the start of HDM challenges (**Figure 2C**).

Significantly more monocytes infiltrated the lungs of HDM-exposed mice compared to PBS-exposed mice following a similar pattern as for eosinophils (**Figure 2D**). These monocytes were in majority Ly6clo, as only few Ly6chi monocytes were found in lung tissue (**Figures 2E, F**). We also investigated monocytes in blood and we found significantly more total and Ly6Chi monocytes in blood of HDM-exposed mice 4 hours after the first sensitization dose (**Supplementary Data, Supplementary Figures 3A–C**). During HDM challenges total, as well as Ly6Chi and Ly6Clo, monocytes increased compared to PBS exposure, culminating in significantly more monocytes at the end of the protocol.

To get an indication of sex differences in the processes we studied, we used both male and female mice. Although the four males and four females per group were too few for rigorous statistical testing, some trends may be identified that could be used to plan for follow-up studies. Female mice tended to have more infiltrating eosinophils in lung tissue than male mice (**Supplementary Data, Supplementary Figure 2A**), which we reported before (10, 25–27), while no clear differences were found for neutrophils and dendritic cells (**Supplementary Data, Supplementary Figures 2B, C**). Similar as found for eosinophils, infiltration of (Ly6Clo) monocytes also tended to be higher in females as compared to males (**Supplementary Data, Supplementary Figures 2D, F**). No clear differences between male and female mice in blood monocytes were found (**Supplementary Data, Supplementary Figures 3D, F**).

### Alveolar macrophages express more CD68, CD11c and YM1 than interstitial macrophages, especially after HDM exposure

**Figure 3:**
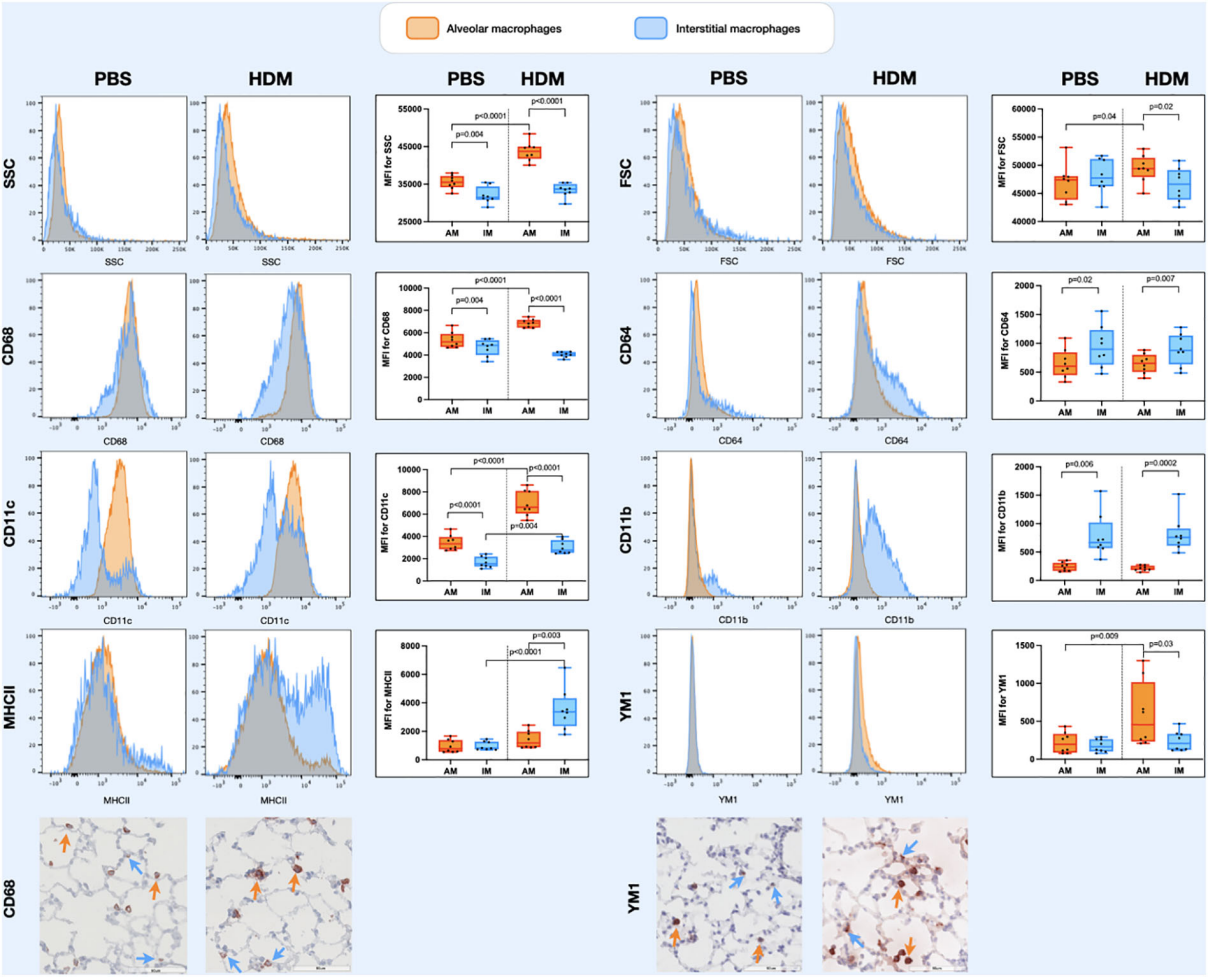
Histograms of forward scatter (FSC), side scatter (SSC), and expression of CD64, CD68, CD11c, CD11b, MHCII and YM1 in alveolar macrophages and interstitial macrophages of both a representative PBS and HDM-exposed mouse from day 11.2 of the model. Graphs to the left of the histograms show quantifications of the mean fluroscence intensity (MFI) of these markers on/in alveolar and interstitial macrophages of all exposed mice. Expression levels on alveolar versus interstitial macrophages were compared using a paired one-way ANOVA with a Sidak’s correction for multiple testing because these cells were isolated from the same animals. Expression levels on alveolar or interstitial macrophages in PBS-exposed animals versus HDM-exposed animals were compared using an unpaired one-way ANOVA with a Sidak’s correction for multiple testing because these cells were isolated from different animals. P-values <0.05 were considered statistically significant. Bottom panels are representative immunohistochemical stainings of CD68 (left) and YM1 (right) of lung tissue of a PBS and a HDM-exposed mouse. Positive cells are stained red and a selection of positive alveolar macrophages are indicated with orange arrows and positive interstitial macrophages with blue arrows.

Populations of lung tissue macrophages (defined as CD68^hi^ cells) were classified based on CD11c and CD11b expression into alveolar macrophages (CD11c^high^CD11b^low^) and interstitial macrophages (CD11c^var^CD11b^var^, see also **Supplementary Data, Supplementary Figures 1**). Using this gating strategy, corrected by ezDAFi (23), we found alveolar macrophages to have more side scatter than interstitial macrophages and to be of similar size when exposed to PBS (**Figure 3**). When exposed to HDM, however, both side and forward scatter increased in alveolar macrophages, while this was not the case for interstitial macrophages. With respect to the different markers we included in our staining, we found that alveolar macrophages had higher expression of CD68 and CD11c and lower expression of CD64 and CD11b than interstitial macrophages. HDM exposure increased CD68 and CD11c expression in alveolar macrophages, while CD64 and CD11b remained stable. Interestingly, YM1 and MHCII expression was similar between alveolar and interstitial macrophages when exposed to PBS, but after HDM exposure MHCII expression specifically increased in interstitial macrophages and YM1 expression in alveolar macrophages. Immunohistochemical staining of lung tissue for CD68 and YM1 confirmed the observations from flow cytometry for alveolar macrophages and interstitial macrophages. We found more intense expression of CD68 and YM1 in lung tissue an HDM mouse compared to a PBS-exposed mouse and an indication that these stainings were more intense in alveolar macrophages than in interstitial macrophages although the latter is harder to appreciate with this immunohistochemical staining (**Figure 3**).

### Lung macrophages peak transiently after HDM challenges start

To study dynamics in the lung tissue macrophage pool during induction of allergic inflammation, we labeled lung macrophages with PKH26 before the start of the experiment. The average labeling efficiency during the model was around 80%, with no significant differences between HDM- and PBS-exposed mice at any time point, suggesting no significant dilution of the pool by incoming unlabeled macrophages during the course of the model (**Supplementary Data Figure 4A**). However, when separating out alveolar and interstitial macrophages some differences became apparent (**Supplementary Data, Supplementary Figures 4B, C**). Both types had around 80% of cells labeled at the start of exposures, but significant dilution of the label was seen for interstitial macrophages in time, especially after a transient increase at the start of HDM challenges in the second week. This suggests slow replenishment by unlabeled cells, possibly incoming monocytes, which was not seen for alveolar macrophages. We also checked other cell types for PKH26 uptake but found no significant presence of this label in monocytes, eosinophils or neutrophils (**Supplementary Data, Supplementary Figures 4D, E**). However, in HDM-exposed mice dendritic cells were found to transiently acquire PKH26-label in the second week of HDM challenges, suggesting uptake of other labeled cells. Furthermore, no clear differences between male and female mice in PKH26-labeling were found (**Supplementary Data, Supplementary Figure 5**)

**Figure 4:**
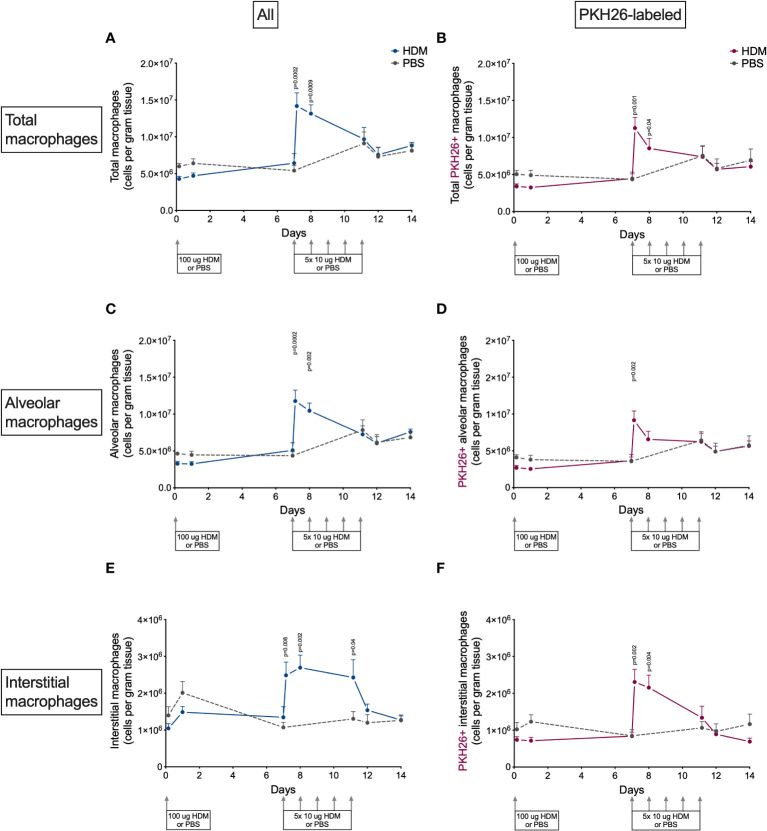
Numbers of **(A)** total macrophages, **(B)** PKH26+ total macrophages, **(C)** alveolar macrophages, D) PKH26+ alveolar macrophages, **(E)** interstitial macrophages, **(F)** PKH26+ interstitial macrophages in lung tissue of house dust mite (HDM)-exposed and phosphate-buffered saline (PBS)-exposed mice at different time points. The differences between HDM and PBS per time point were tested using a Student’s t test. Day 7.2 and day 8 of HDM were compared to day 7 of PBS with a one-way ANOVA with Sidak’s correction for multiple testing because these mice did not have matching PBS-exposed mice. P<0.05 was considered significant. Per time point the geometric mean and standard error of the mean per group are shown (4 males and 4 females per group, except for PBS 14 days that had 3 males and 4 females).

The presence of macrophages in lung tissue after exposure to HDM showed an interesting pattern (**Figure 4**). The first week after HDM sensitization total macrophage numbers did not differ between HDM and PBS-exposed mice, but they increased dramatically four hours and one day after the first of the HDM challenges. Numbers returned to PBS-levels thereafter for the remainder of the HDM challenges. Both alveolar and interstitial macrophages followed that same pattern, both contributing to the transient peak in macrophages. However, the peak in interstitial macrophages was one day longer than for alveolar macrophages. We found no obvious differences between male and female mice (**Supplementary Data, Supplementary Figure 6**).

To investigate if this peak was the results of proliferation of local macrophages or infiltration of monocytes, we also determined the number of PKH26-labeled macrophages in lung tissue. PKH26-labeled macrophages showed a similar pattern as nonlabeled macrophages (**Figure 4**), albeit somewhat lower in general, partly caused by the 80% labeling efficiency and partly by fewer labeled interstitial macrophages. This suggests that both local proliferation (mostly of alveolar macrophages) and possibly infiltration of monocytes (mostly adding to interstitial macrophages) contribute to the increase in lung macrophages after starting HDM challenges.

### YM1+ macrophages develop locally, while MHCII-hi macrophages appear to be derived from incoming monocytes

We then investigated the distribution of the different polarization states, taking both alveolar and interstitial macrophages together first (**Supplementary Data, Supplementary Figure 7**). At baseline, most macrophages had a nonpolarized phenotype (CD68^hi^MHCII^low^YM1-; hereafter named MHCII-loYM1-) and only few were polarized towards a Th1-associated phenotype (CD68^hi^MHCII^hi^; hereafter named MHCII-hi) or a Th2-associated phenotype (CD68^hi^MHCII^lo^YM1+; hereafter named YM1+).

In the first week after HDM sensitization no substantial changes in polarization were observed compared to PBS-exposed mice. However, numbers of nonpolarized macrophages increased dramatically when HDM challenges started in the second week (**Supplementary Data, Supplementary Figure 7A**) and were mostly driven by local proliferation as most unpolarized macrophages were PKH26 labeled (**Supplementary Data, Supplementary Figure 7B**). This increase was only temporary because numbers of nonpolarized macrophages then plummeted significantly below those of PBS-exposed animals. At the same time in this second week, numbers of MHCII-hi macrophages increased transiently (**Supplementary Data, Supplementary Figure 7C**) and this was not driven by local macrophages as there was no concomitant increase in PKH26-labeled macrophages (**Supplementary Data, Supplementary Figure 7D**). YM1+ macrophages increased profoundly (**Supplementary Data, Supplementary Figure 7E**) and in contrast to MHCII-hi macrophages, these YM1+ macrophages were all of local origin (**Supplementary Data, Supplementary Figure 7F**).

As we described before, female mice tended have higher numbers of MHCII-hi and YM1+ macrophages than males (**Supplementary Data, Supplementary Figure 8**) (10, 25).

**Figure 5:**
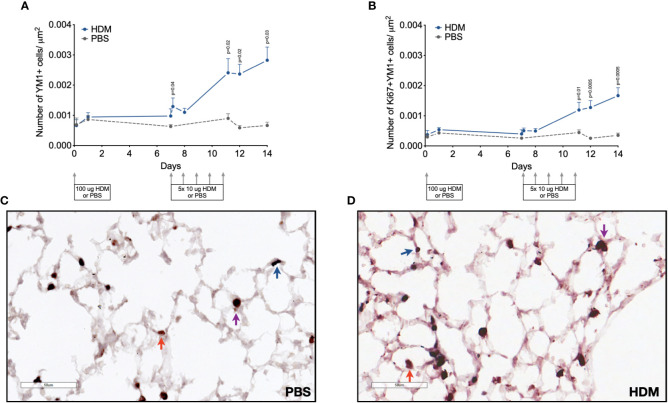
Numbers of **(A)** YM1+ macrophages, and **(B)** proliferating Ki67+ YM1+ cells in lung tissue of house dust mite (HDM)-exposed and phosphate-buffered saline (PBS)-exposed mice at different time points during exposure. The differences between HDM and PBS per time point were tested using a Student’s t test. Day 7.2 and day 8 of HDM were compared to day 7 of PBS with a one-way ANOVA with Sidak’s correction for multiple testing because these mice did not have matching PBS-exposed mice. P<0.05 was considered significant. Per time point the geometric mean and standard error of the mean per group are shown (4 males and 4 females per group, except for PBS 14 days that had 3 males and 4 females).Panels **(C, D)** are representative pictures of the YM1 (red) and Ki67(blue) double stainings of lung tissue of a PBS **(C)** and a HDM-exposed **(D)** mouse. Examples of YM1-single positive cells are indicated with red arrows, Ki67-single positive cells with blue arrows, and YM1/Ki67 double positive cells with purple arrows.

Since YM1+ macrophages were found to make up an important part of macrophages that increase during HDM challenges and were mostly PKH26+, we confirmed their presence and possible proliferation in lung tissue by immunohistochemistry. Immunohistochemical analysis of the total number of YM1+ macrophages in lung tissue showed a similar curve to the one obtained by flow cytometry (compare **Figure 5A** with supplemental **Supplementary Figure 7E**) with low numbers in the first week after HDM sensitization and a steady increase in YM1+ macrophages in the second week during HDM challenges. Female mice tended to have higher numbers of YM1+ macrophages than males again (**Supplementary Data, Supplementary Figure** 9). We then investigated whether the increase in YM1+ macrophages was the result of proliferation by costaining for Ki67, a marker of proliferating cells. The number of YM1+Ki67+ cells followed a similar pattern to that observed for YM1+ macrophages albeit at a lower level, suggesting part of the increase was driven by proliferation (**Figure 5B**).

Together, these data suggest the transient peak in macrophages is driven by local proliferation of unpolarized macrophages that then polarize towards YM1+ macrophages. The increase in YM1+ macrophages is driven by both unpolarized macrophages acquiring YM1 and proliferation of these YM1+ macrophages, while the increase in MHCII-hi macrophages appears to be driven by differentiating incoming monocytes.

### Alveolar macrophages are mostly YM1+ while interstitial macrophages preferentially upregulate MHCII

**Figure 6:**
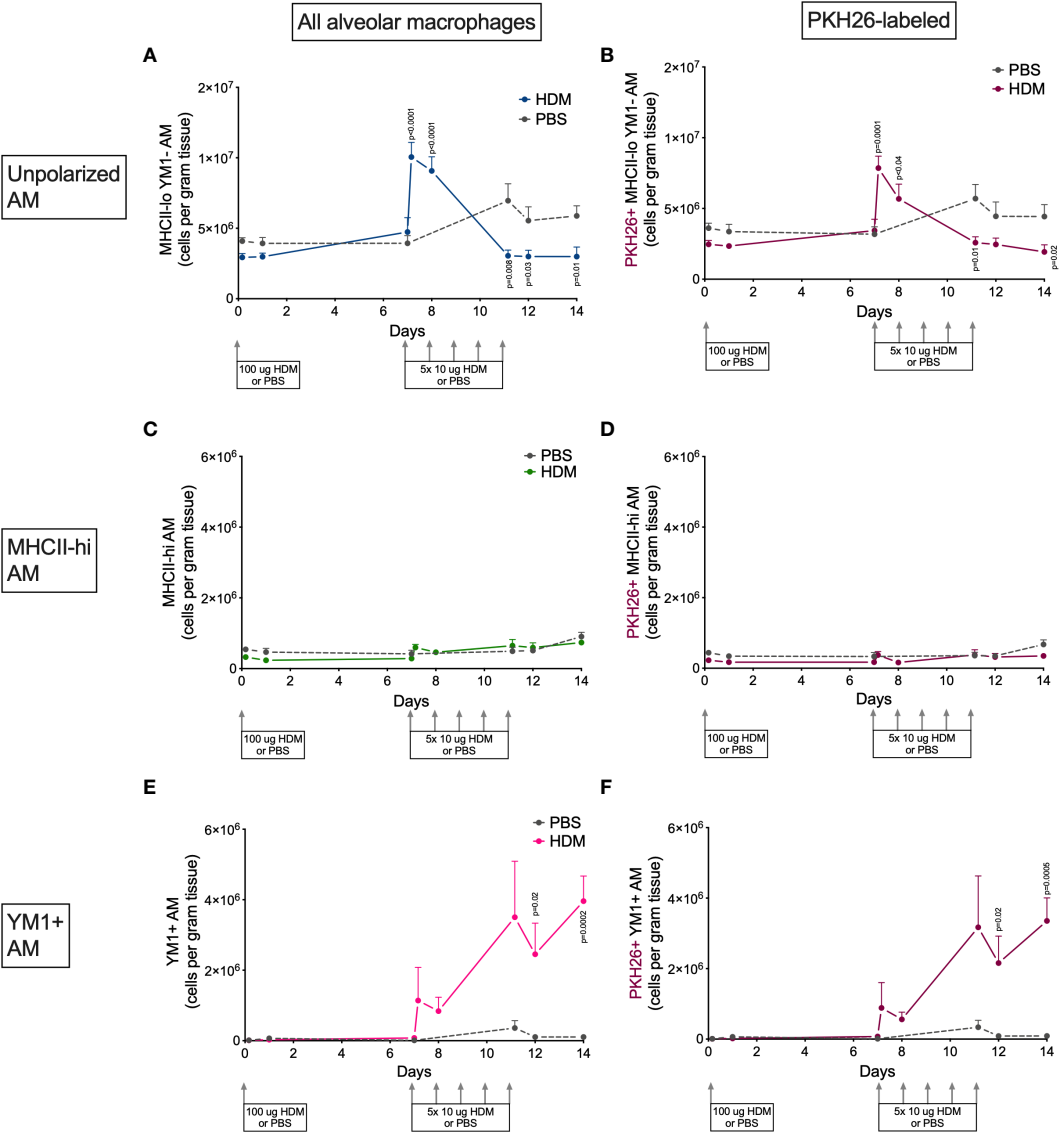
Numbers of **(A)** MHCII-loYM1- alveolar macrophages, **(B)** PKH26+MHCII-loYM1-alveolar macrophages, **(C)** MHCII-hi alveolar macrophages, **(D)** PKH26+MHCII-hi alveolar macrophages, E) YM1+ alveolar macrophages, **(F)** and PKH26+YM1+ alveolar macrophages in lung tissue of house dust mite (HDM)-exposed and phosphate-buffered saline (PBS)-exposed mice at different time points during exposure. The differences between HDM and PBS per time point were tested using a Student’s t test. Day 7.2 and day 8 of HDM were compared to day 7 of PBS with a one-way ANOVA with Sidak’s correction for multiple testing because these mice did not have matching PBS-exposed mice. P<0.05 was considered significant. Per time point the geometric mean and standard error of the mean per group are shown (4 males and 4 females per group, except for PBS 14 days that had 3 males and 4 females).

**Figure 7:**
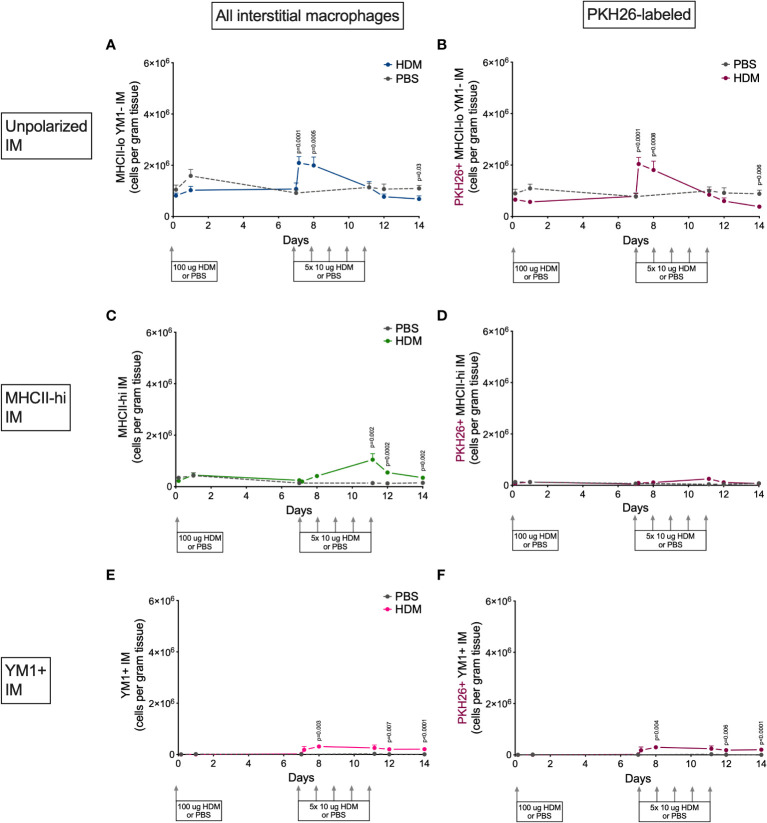
Numbers of **(A)** MHCII-loYM1- interstitial macrophages, **(B)** PKH26+MHCII-loYM1- interstitial macrophages, **(C)** MHCII-hi interstitial macrophages, **(D)** PKH26+MHCII-hi interstitial macrophages, **(E)** YM1+ interstitial macrophages, **(F)** and PKH26+YM1+ interstitial macrophages in lung tissue of house dust mite (HDM)-exposed and phosphate-buffered saline (PBS)-exposed mice at different time points during exposure. The differences between HDM and PBS per time point were tested using a Student’s t test. Day 7.2 and day 8 of HDM were compared to day 7 of PBS with a one-way NoANOVA with Sidak’s correction for multiple testing because these mice did not have matching PBS-exposed mice. P<0.05 was considered significant. Per time point the geometric mean and standard error of the mean per group are shown (4 males and 4 females per group, except for PBS 14 days that had 3 males and 4 females).

Alveolar and interstitial macrophages have different functions adapted to their location in tissue (1). We therefore investigated if polarization differed between these two types of lung macrophages. Both alveolar and interstitial macrophages were in a nonpolarized state at the beginning of the model and started to polarize after the first challenge dose with HDM (**Figures 6** and **7**). However, during HDM challenges alveolar macrophages polarized towards a YM1+ phenotype and did not upregulate MHCII (**Figure 6**), whereas interstitial macrophages preferentially polarized towards an MHCII-hi phenotype (**Figure 7**). The PKH26 staining revealed that YM1+ alveolar and interstitial macrophages developed from local macrophages, while MHCII-hi interstitial macrophages did not. Again, female mice tended have higher percentages of MHCII-hi and YM1+ alveolar and interstitial macrophages than males (**Supplementary Data, Supplementary Figures 10, 11**).

## Discussion

This study investigated the kinetics of polarization responses of both alveolar and interstitial lung macrophages to allergen exposure in mice. In a 14-day model of HDM exposure, we have shown that YM1+ and MHCII+ polarization starts up in the second week of HDM exposures. Before this happened, nonpolarized macrophages transiently increased in HDM-exposed mice. This transient increase was mostly local proliferation of alveolar macrophages, while interstitial macrophages were also expanded with unlabeled macrophages suggesting monocyte contribution. Although we found high proliferation of YM1+ macrophages, it did not account for the full increase in YM1+ alveolar macrophages and these seem to develop by polarization of nonpolarized macrophages. Our results suggest that the lung macrophage population reacts dynamically to an allergen threat: first with proliferation of both nonpolarized alveolar and interstitial macrophages and recruitment of monocyte-derived interstitial macrophages and then polarization of these macrophages.

A key feature of allergic lung inflammation is the rapid accumulation of innate immune cells that are recruited from blood. The rapid influx of granulocytes and monocytes (the latter being possible precursors for dendritic cells and macrophages) are prominent in this cascade (28). Interestingly, we found that neutrophils were the first granulocytes that infiltrated lung tissue after sensitization to HDM rather than eosinophils or monocytes, which infiltrated the lung only after HDM sensitization and challenge. These findings are in line with previous studies that also investigated the kinetics of eosinophils versus neutrophils in a rat and mouse model of ovalbumin-induced allergic lung inflammation and confirm allergic inflammation was successfully induced (29, 30).

The main focus of our study was macrophage kinetics during HDM-induced lung inflammation. We found that, although the total number of macrophages at the end of the model did not differ between HDM exposure and PBS exposure, a clear peak in total macrophages was seen in week two when the lungs were challenged to HDM. We found the same peak for alveolar and interstitial macrophages and these macrophages were mostly PKH26+ in case of alveolar macrophages and less so in the case of interstitial macrophages. These increases were not due to an artefact of having sacrificed certain groups on one day, as we staggered our experiments and therefore each experimental group consisted of animals sacrificed on 4 different days. Our data therefore strongly suggest that the peak in macrophages during HDM challenges must be due to proliferating local alveolar and interstitial macrophages, while interstitial macrophages are also expanded with recruited monocytes. This confirms the importance of proliferation described earlier by Zaslona et al. who had similar findings with HDM (19). Their study, however, did not track interstitial and alveolar macrophages and we now show that both contribute to the transient increase in macrophages during HDM challenges and that monocytes do appear to contribute to the pool of interstitial macrophages. Interestingly, the peak in interstitial macrophages is mirrored by a peak in (Ly6Chi) monocytes in blood, possibly suggesting these may be used to expand the interstitial macrophage pool during that peak, as was shown for steady-state conditions before (17, 31–33). It is impossible to tell, which type of monocytes, Ly6Chi, Ly6Clo, or both, contributes to the overall pool of interstitial macrophages after HDM exposures from our data. Both have been shown to replenish subsets of interstitial macrophages (17, 31, 32). We did also find significant infiltration of Ly6Clo monocytes into lung tissue during HDM challenges while Ly6Chi monocytes were not present at all, suggesting these Ly6Clo monocytes may be the one transitioning to interstitial macrophages as has been described by Schyns et al. (32). They showed that CD64+CD16.2+ monocytes arise from intravascular Ly-6Clo patrolling monocytes that enter lung tissue at steady-state to become interstitial macrophages. However, we cannot exclude the possibility that Ly6Chi monocytes extravasate and quickly lose their Ly6C expression as described for migratory monocytes that downregulate Ly6C upon tissue entry and ultimately migrate to lung-draining lymph nodes to present antigen (34).

To improve our gating strategy for the different myeloid cells, we used a novel directed unsupervised clustering approach called ezDAFi to correct our manual gating with the multidimensional data available for each event (23). Although the manual gating strategy is used, the results of ezDAFi are data-driven by unsupervised clustering methods and this hugely improved separation of cell populations, especially of alveolar and interstitial macrophages. Our results also compare well with others who have looked into the differences of these two populations of macrophages in healthy conditions in mice (17, 18, 32, 35, 36).

Tracking the different polarization states during HDM exposure was another important aim of our study. The apparent nonresponse in the first week rapidly changed after the first challenge with 10 μg HDM with a significant peak in nonpolarized macrophages, quickly followed by an almost complete loss of nonpolarized macrophages and by increases in polarized macrophages. Our data suggest that local macrophages first proliferate and then follow a polarization program. Most of the local alveolar macrophages polarize towards YM1 positivity, while infiltrating monocytes seem to supply the pool of MHCII-hi interstitial macrophages.

This divergent development of MHCII-hi interstitial macrophage and YM1+ alveolar macrophages in response to HDM is interesting and suggests functional differences. No clear data exist on explaining the different responses, but we speculate that interstitial macrophages are better placed to interact with T lymphocytes and therefore upregulate MHCII. This notion is confirmed by findings from two studies showing MHCII-hi interstitial macrophages in lung tissue that have immune regulatory functions requiring high expression of MHCII (17, 33). In contrast, alveolar macrophages are situated in the air spaces, with direct contact with the outside world, and therefore could have more use for YM1. The function of YM1 remains elusive but it has clear associations with stimulating clearance of helminths and allergens (37), both of which enter through the airspaces.

In conclusion, in a 14-day HDM model we have shown that development of a polarized macrophage pool during allergic inflammation is first dependent on proliferation of nonpolarized tissue-resident macrophages with some help of infiltrating unlabeled cells, presumably circulating monocytes. After supplementation of nonpolarized macrophages, both alveolar and interstitial macrophages polarize at the cost of their nonpolarized counterparts during HDM challenges. These were mostly YM1+ alveolar macrophages and MHCII-hi interstitial macrophages. This novel information will help us to better understand the role of macrophages in asthma and designing therapeutic strategies targeting macrophage functions.

## Data availability statement

The raw data supporting the conclusions of this article will be made available by the authors, without undue reservation.

## Ethics statement

The animal study was reviewed and approved by The Institutional Animal Care and Use Committee of the University of Groningen, Groningen, Netherlands.

## Author contributions

CD and BM designed and planned the experiments. CD, LF-S, CR-S, EP and FD conducted mouse studies and laboratory experimental work. BM analyzed results and wrote the manuscript. All authors contributed to the article and approved the submitted version.
