# Supplementary material for: Explaining the polarized macrophage pool during murine allergic lung inflammation
Source: Front Immunol. 2022 Dec 20;13:1056477. doi: 10.3389/fimmu.2022.1056477 (PMC9807907; doi:10.3389/fimmu.2022.1056477)
Supplement: Supplementary file 1 [file DataSheet_1.pdf]

Supplementary data to:

Explaining the polarized macrophage pool during murine allergic lung inflammation

Christina Draijer[1*], Laura Florez-Sampedro[1, 2, 3*], Catharina Reker-Smit[4], Eduard Post[4], Fransien van Dijk[1,3], Barbro N. Melgert[1,3]

[1] University of Groningen, University Medical Center Groningen, GRIAC- Groningen Research Institute for Asthma and COPD, Groningen, The Netherlands
[2] University of Groningen, Department of Chemical and Pharmaceutical Biology, Groningen, The Netherlands
[3] University of Groningen, Department of Molecular Pharmacology, Groningen, The Netherlands
[4] University of Groningen, Department of Pharmacokinetics, Toxicology and Targeting, Groningen, The Netherlands

*These authors contributed equally to this work

Corresponding author:
Prof Dr B.N. Melgert
Department of Molecular Pharmacology
University of Groningen
Antonius Deusinglaan 1
9713 AV Groningen
The Netherlands
Tel: +31-6-31921664
Email: b.n.melgert@rug.nl

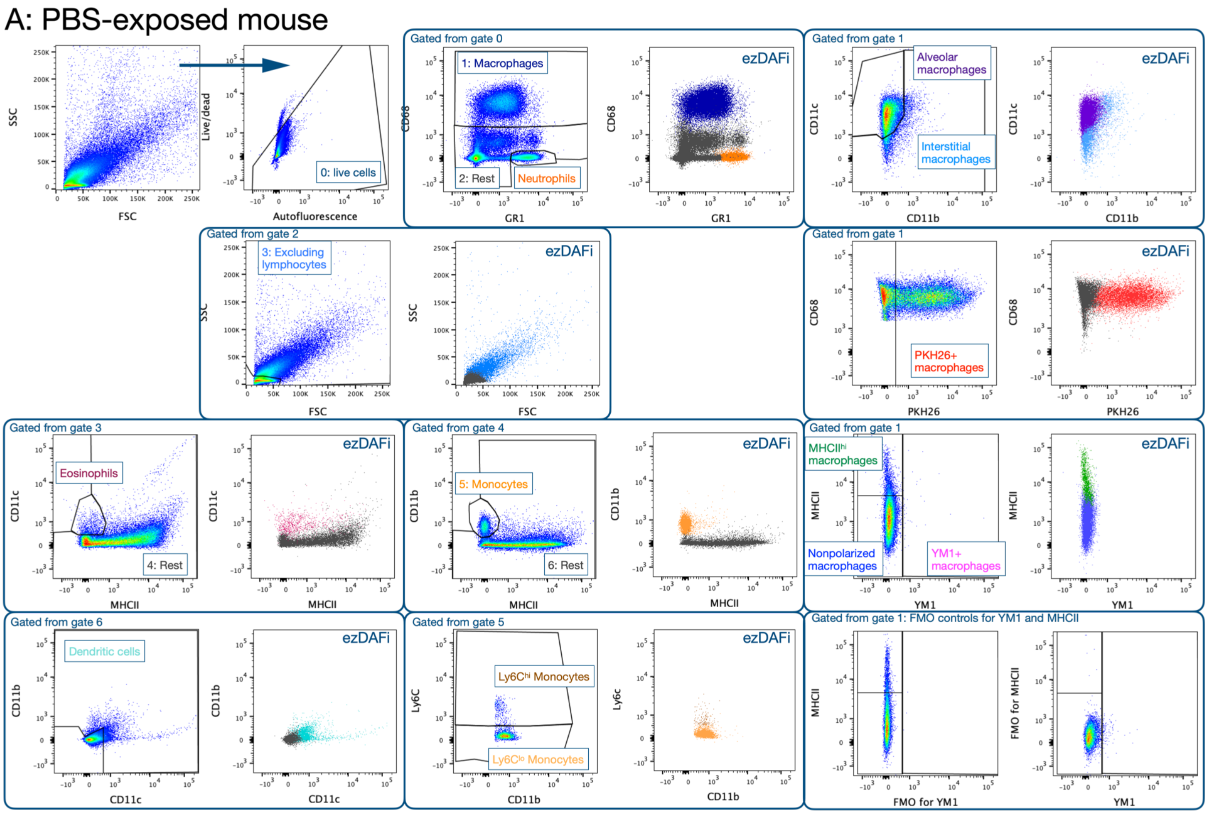

Supplementary figure 1A. Gating strategy of macrophages, neutrophils, eosinophils, dendritic cells and monocytes in lung tissue of a PBS-exposed mouse. PKH26 labeling is shown for total macrophages and represents the gating performed for both interstitial and alveolar macrophage subtypes. Macrophage polarization is shown for total macrophages and represents the gating performed for alveolar and interstitial macrophages. Original gates are shown on the left of each panel and populations corrected by ezDAFi are shown on the right.

B: HDM-exposed mouse

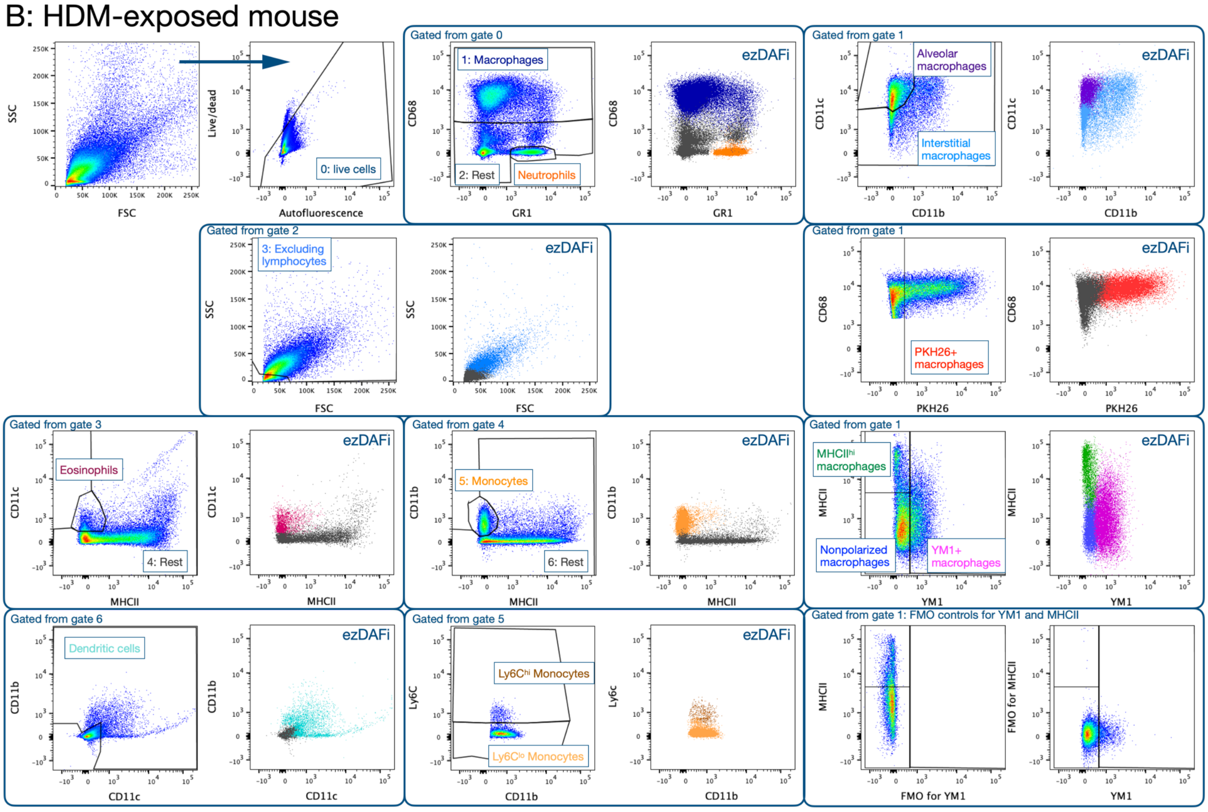

Supplementary figure 1B. Gating strategy of macrophages, neutrophils, eosinophils, dendritic cells and monocytes in lung tissue of an HDM-exposed mouse. PKH26 labeling is shown for total macrophages and represents the gating performed for both interstitial and alveolar macrophage subtypes. Macrophage polarization is shown for total macrophages and represents the gating performed for alveolar and interstitial macrophages. Original gates are shown on the left of each panel and populations corrected by ezDAFi are shown on the right.

C: Gating of blood monocytes

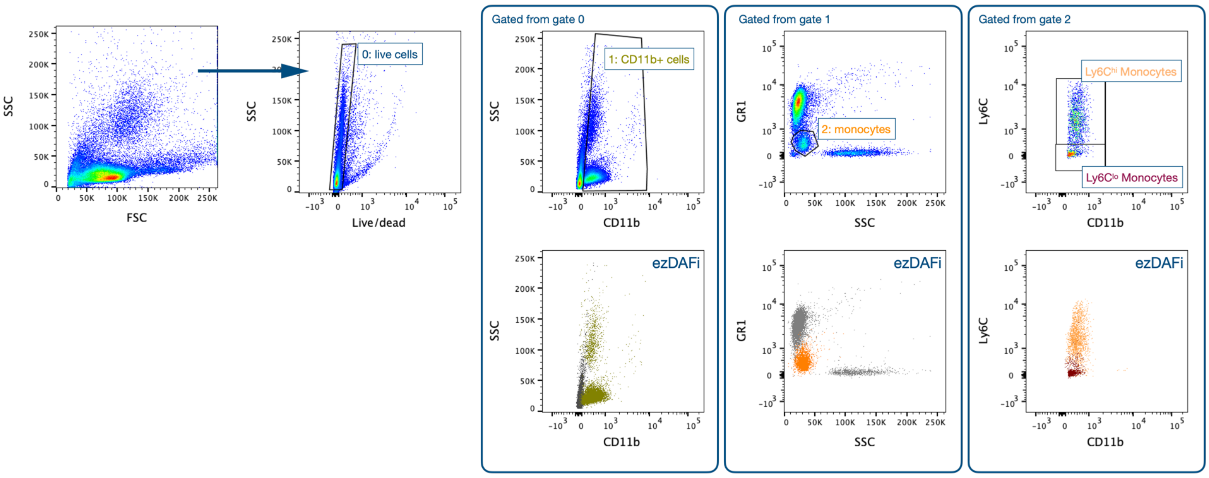

Supplementary figure 1C. Gating strategy for blood monocytes. Original gates are shown on the left of each panel and populations corrected by ezDAFi are shown on the right.

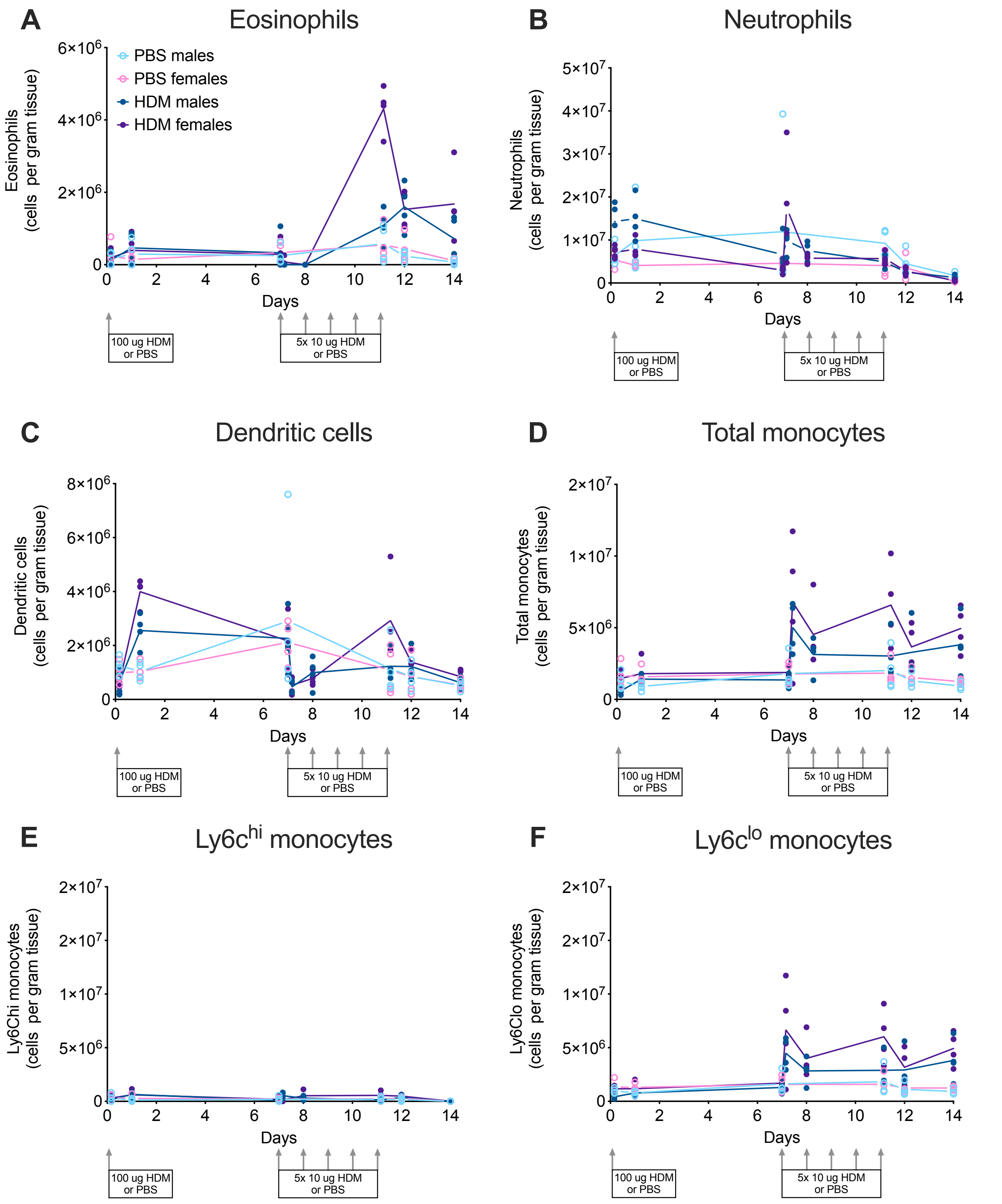

Supplementary figure 2. Numbers of **A)** eosinophils, **B)** neutrophils, **C)** dendritic cells, **D)** total monocytes, **E)** Ly6Chi monocytes, and **F)** Ly6Clo monocytes in lung tissue of house dust mite (HDM)-exposed and phosphate-buffered saline (PBS)-exposed mice at different time points separated out for males and females. Each individual mouse is a dot.

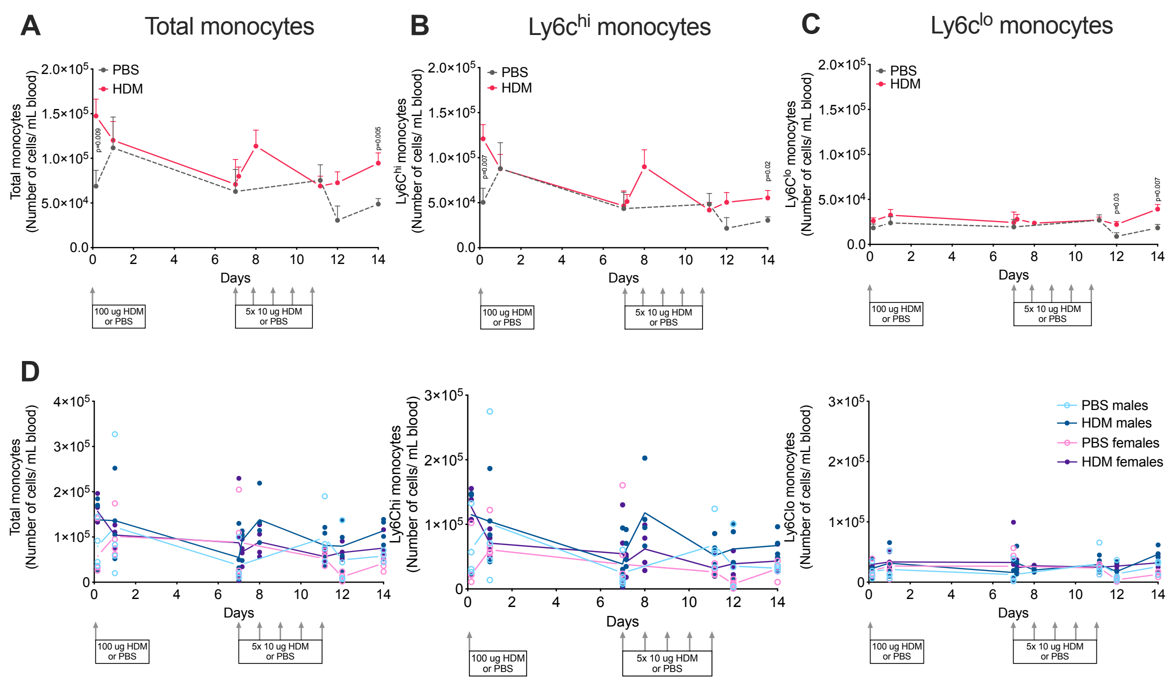

Supplementary figure 3. Total number of **A)** blood monocytes, **B)** Ly6Chi blood monocytes, and **C)** and Ly6Clo blood monocytes per mL blood of HDM-exposed and PBS-exposed mice at different time points of the 14-day model. The differences between HDM and PBS per time point were tested using a Student's t test. Day 7.2 and day 8 of HDM were compared to day 7 of PBS with a one-way ANOVA with Sidak's correction for multiple testing because these mice did not have matching PBS-exposed mice. P<0.05 was considered significant. Per time point the geometric mean and standard error of the mean per group are shown (4 males and 4 females per group, except for PBS 14 days that had 3 males and 4 females).

Total number of **D)** blood monocytes, **E)** Ly6Chi blood monocytes, and **F)** and Ly6Clo blood monocytes per mL blood of HDM-exposed and PBS-exposed mice at different time points of the 14-day HDM model separated out for males and females. Each individual mouse is a dot.

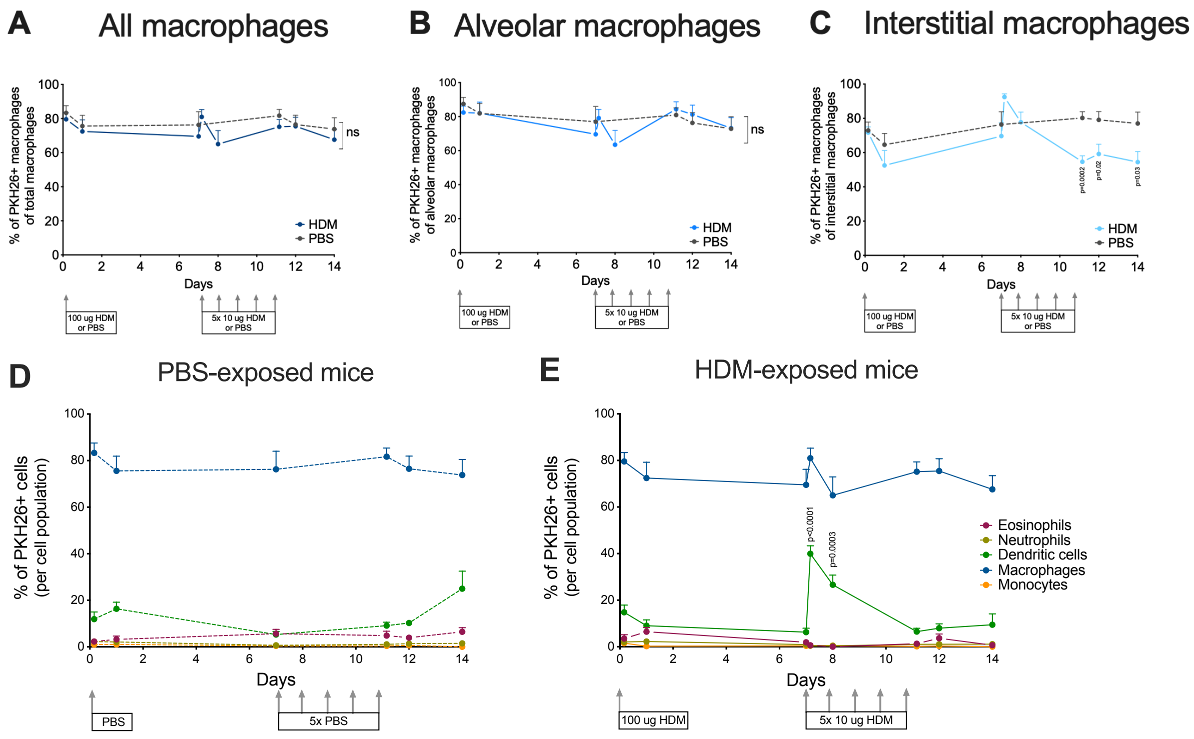

Supplementary figure 4. Labeling efficiency of **A)** all macrophages, **B)** alveolar macrophages, **C)** interstitial macrophages in lung tissue of house dust mite (HDM)-exposed and phosphate-buffered saline (PBS)-exposed mice at different time points during exposure. The differences between HDM and PBS per time point were tested using a Student's t test. Day 7.2 and day 8 of HDM were compared to day 7 of PBS with a one-way ANOVA with Sidak's correction for multiple testing because these mice did not have matching PBS-exposed mice. P<0.05 was considered significant. Per time point the geometric mean and standard error of the mean per group are shown (4 males and 4 females per group, except for PBS 14 days that had 3 males and 4 females).

Percentages of PKH26-labeled myeloid cells in **D)** phosphate-buffered saline (PBS)-exposed and **E)** house dust mite (HDM)-exposed mice at different time points of the 14-day HDM model. The differences between HDM and PBS were tested using a Student's t test, per time point. Day 7.2 and day 8 of HDM were compared to day 7 of PBS with a one-way ANOVA with Sidak's correction for multiple testing. P<0.05 was considered significant. Per time point the geometric mean and standard error of the mean per group are shown (4 males and 4 females per group, except for PBS 14 days that had 3 males and 4 females).

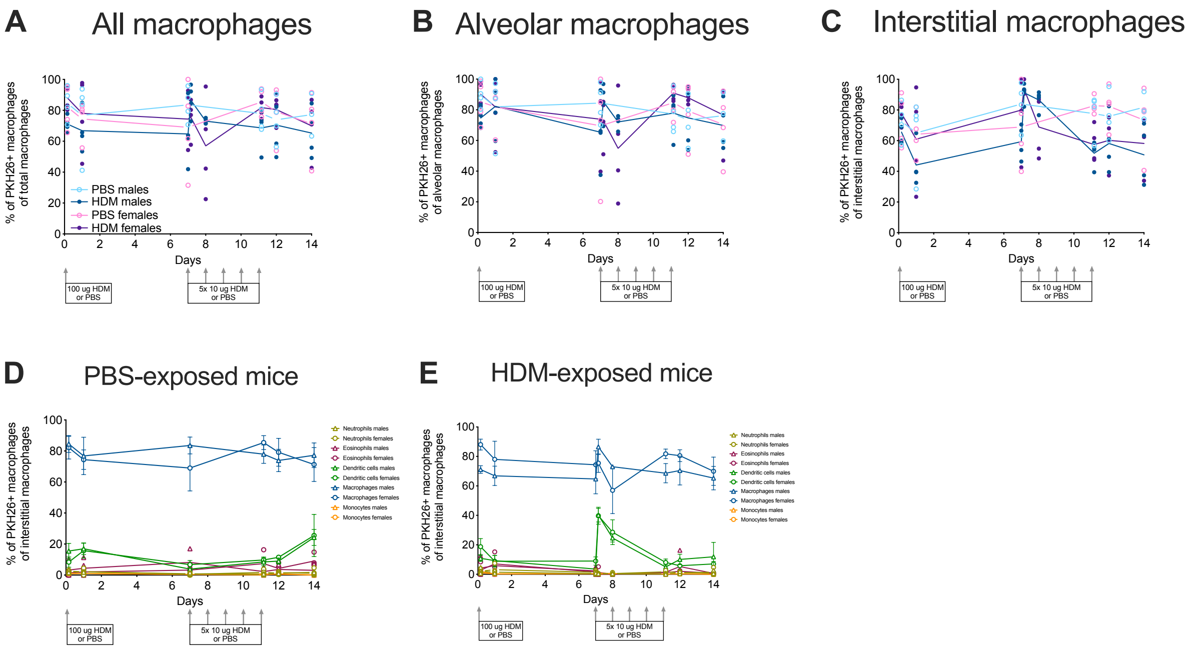

Supplementary figure 5. Labeling efficiency of **A)** all macrophages, **B)** alveolar macrophages, **C)** interstitial macrophages in lung tissue of house dust mite (HDM)-exposed and phosphate-buffered saline (PBS)-exposed mice at different time points during exposure, separated out for males and females. Each individual mouse is a dot.

Percentages of PKH26-labeled myeloid cells in **D)** phosphate-buffered saline (PBS)-exposed and **E)** house dust mite (HDM)-exposed mice at different time points of the 14-day HDM model, separated out for males and females. Per time point the geometric mean and standard error of the mean per group of males (triangles) and females (circles) are shown (4 males and 4 females per group, except for PBS 14 days that had 3 males and 4 females).

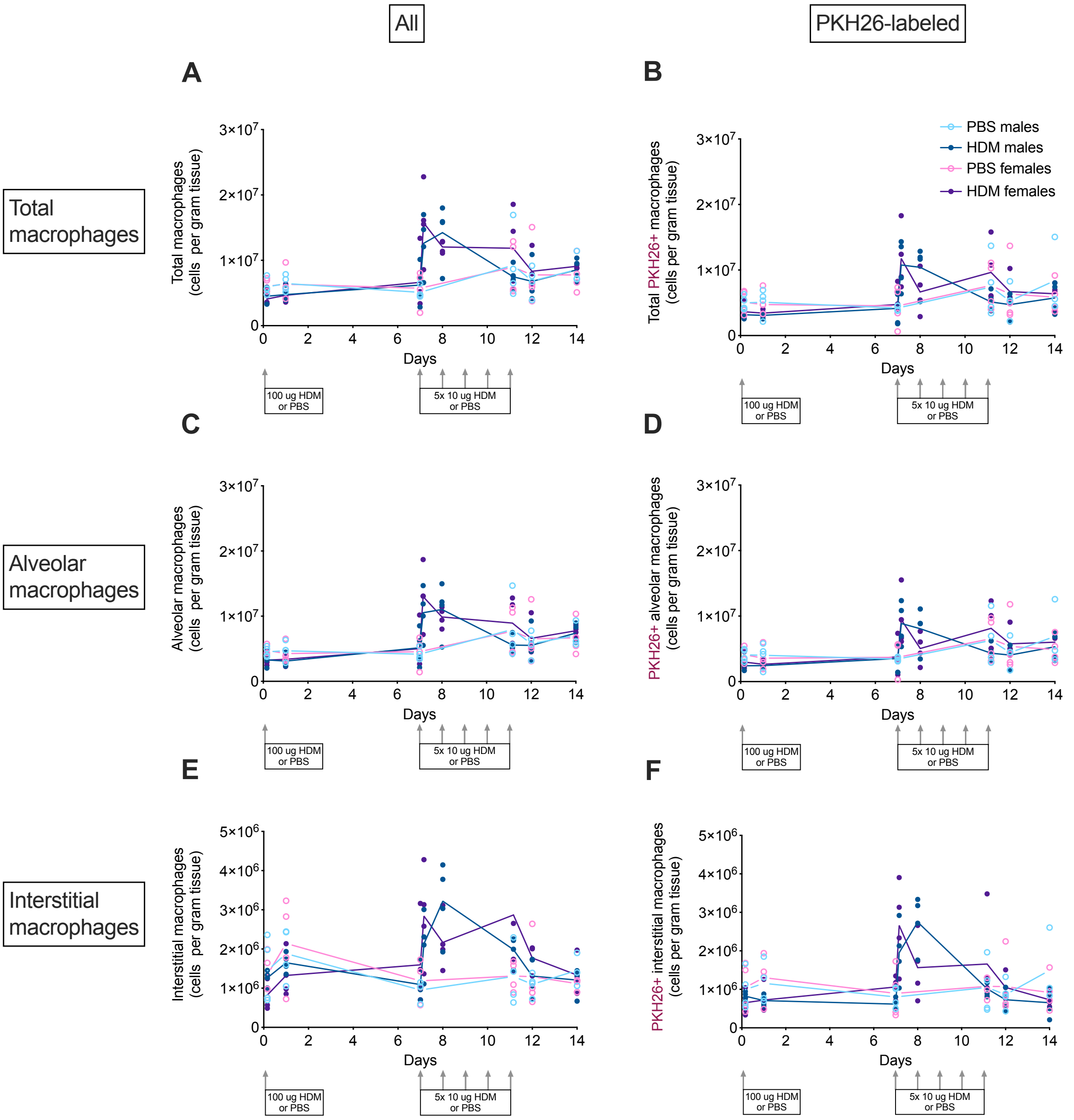

Supplementary figure 6. Numbers of **A)** total macrophages, **B)** PKH26+ total macrophages, **C)** alveolar macrophages, **D**) PKH26+ alveolar macrophages, **E)** interstitial macrophages, **F)** PKH26+ interstitial macrophages in lung tissue of house dust mite (HDM)-exposed and phosphate-buffered saline (PBS)-exposed mice at different time points during exposure separated out for males and females. Each individual mouse is a dot.

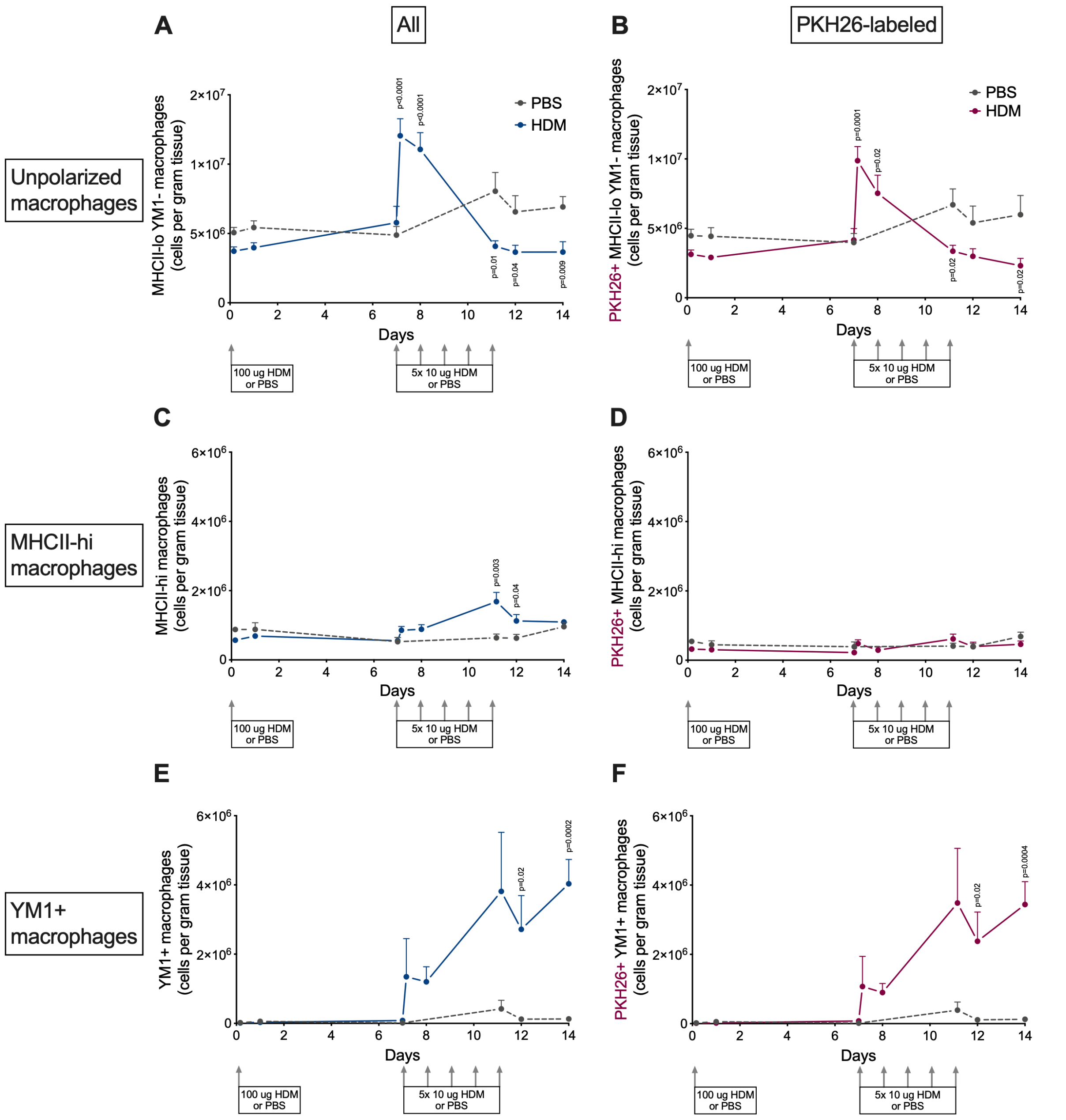

Supplementary figure 7. Numbers of **A)** MHCII-loYM1- macrophages, **B)** PKH26+MHCII-loYM1- macrophages, **C)** MHCII-hi macrophages, **D)** PKH26+MHCII-hi macrophages, **E)** YM1+ macrophages, **F)** and PKH26+YM1+ macrophages in lung tissue of house dust mite (HDM)-exposed and phosphate-buffered saline (PBS)-exposed mice at different time points during exposure. The differences between HDM and PBS per time point were tested using a Student's t test. Day 7.2 and day 8 of HDM were compared to day 7 of PBS with a one-way ANOVA with Sidak's correction for multiple testing because these mice did not have matching PBS-exposed mice. P<0.05 was considered significant. Per time point the geometric mean and standard error of the mean per group are shown (4 males and 4 females per group, except for PBS 14 days that had 3 males and 4 females).

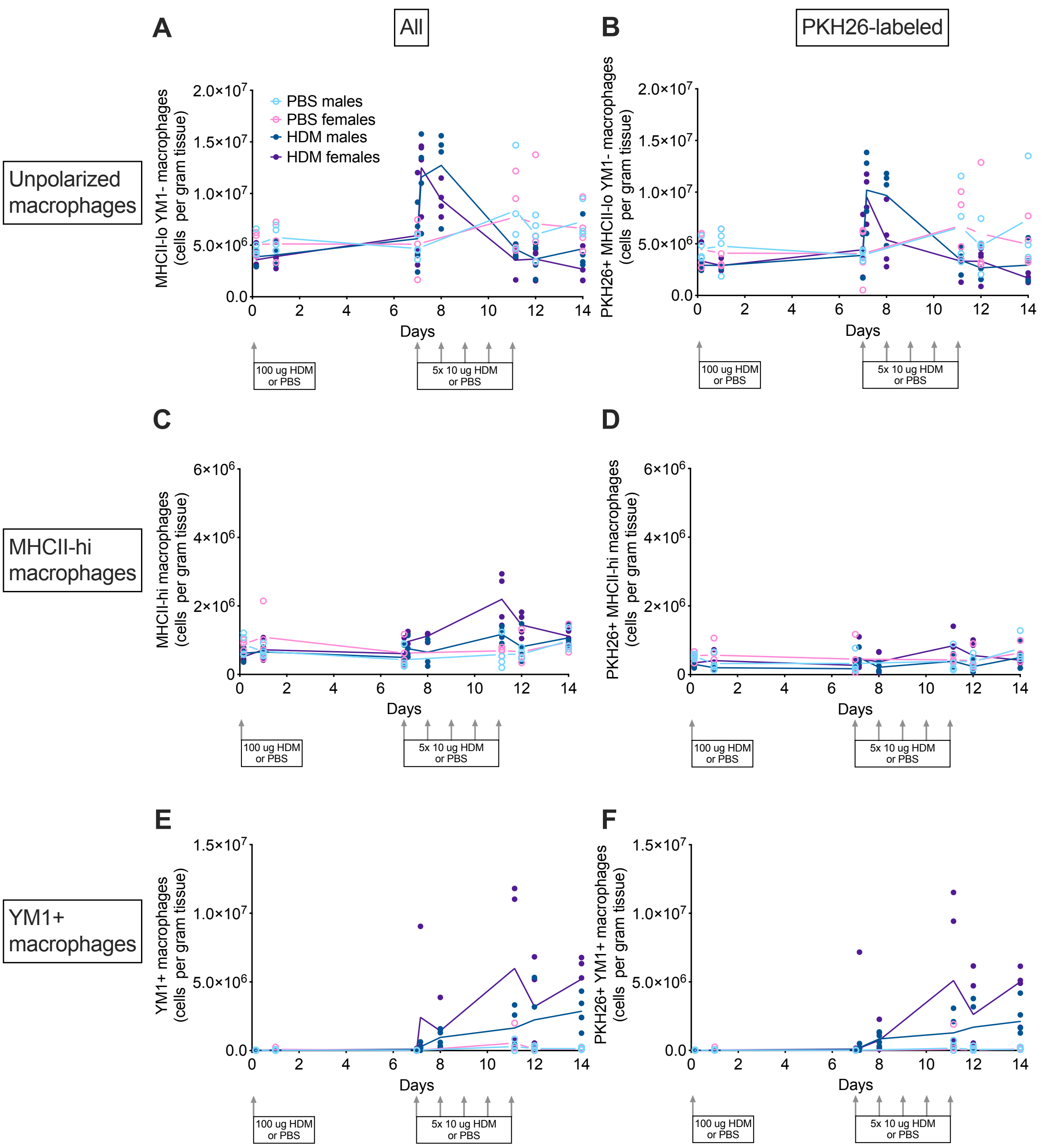

Supplementary figure 8. Numbers of **A)** MHCII-loYM1- macrophages, **B)** PKH26+MHCII-loYM1- macrophages, **C)** MHCII-hi macrophages, **D)** PKH26+MHCII-hi macrophages, **E)** YM1+ macrophages, **F)** and PKH26+YM1+ macrophages in lung tissue of house dust mite (HDM)-exposed and phosphate-buffered saline (PBS)-exposed mice at different time points during exposure separated out for males and females. Each individual mouse is a dot.

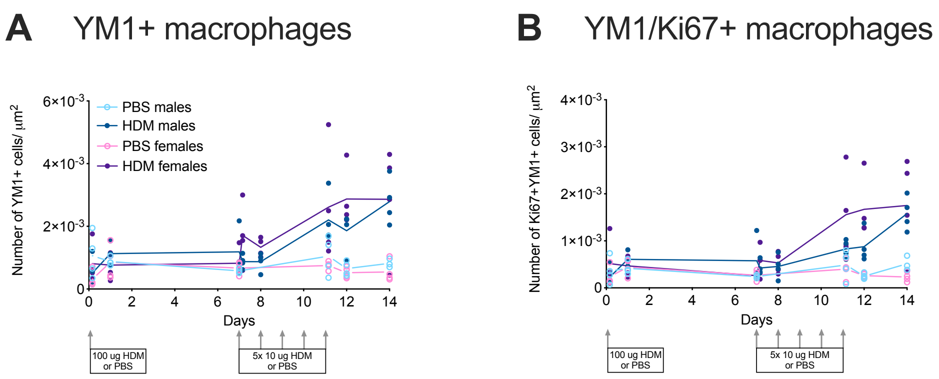

Supplementary figure 9. Numbers of **A)** YM1+ macrophages, and **B)** proliferating Ki67+ YM1+ cells in lung tissue of house dust mite (HDM)-exposed and phosphate-buffered saline (PBS)-exposed mice at different time points during exposure separated out for males and females. Each individual mouse is a dot.

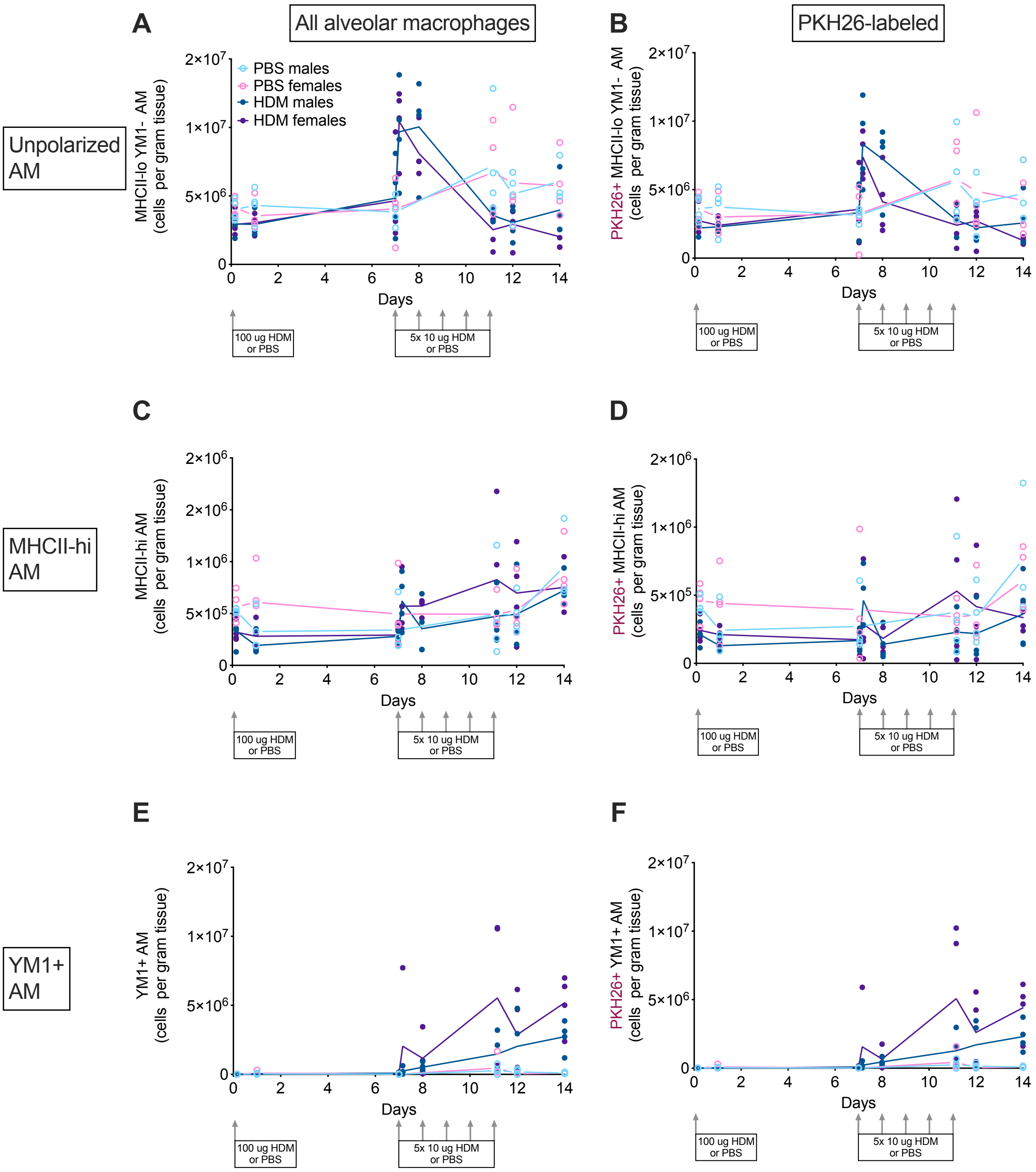

Supplementary figure 10. Numbers of **A)** MHCII-loYM1- alveolar macrophages, **B)** PKH26+MHCII-loYM1-alveolar macrophages, **C)** MHCII-hi alveolar macrophages, **D)** PKH26+MHCII-hi alveolar macrophages, **E)** YM1+ alveolar macrophages, **F)** and PKH26+YM1+ alveolar macrophages in lung tissue of house dust mite (HDM)-exposed and phosphate-buffered saline (PBS)-exposed mice at different time points during exposure separated out for males and females. Each individual mouse is a dot.

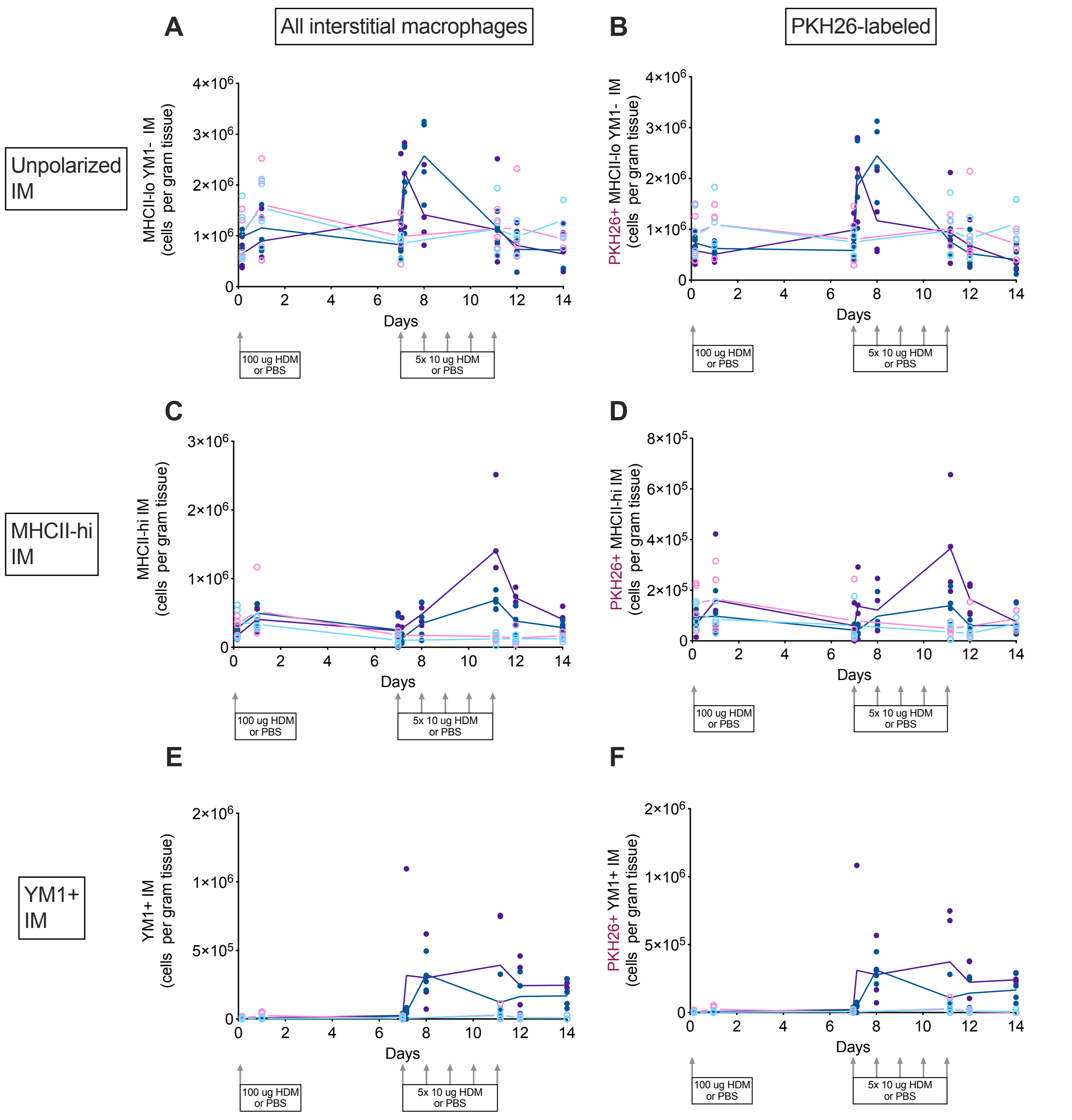

Supplementary figure 11. Numbers of **A)** MHCII-loYM1- interstitial macrophages, **B)** PKH26+MHCII-loYM1- interstitial macrophages, **C)** MHCII-hi interstitial macrophages, **D)** PKH26+MHCII-hi interstitial macrophages, **E)** YM1+ interstitial macrophages, **F)** and PKH26+YM1+ interstitial macrophages in lung tissue of house dust mite (HDM)-exposed and phosphate-buffered saline (PBS)-exposed mice at different time points during exposure separated out for males and females. Each individual mouse is a dot.
